# Sampling plan and methodological aspects: a household healthcare survey in Piauí

**DOI:** 10.11606/s1518-8787.2021055003441

**Published:** 2021-12-09

**Authors:** Lays Arnaud Rosal Lopes Rodrigues, Danilla Michelle Costa e Silva, Edina Araújo Rodrigues Oliveira, Layanne Cristina de Carvalho Lavôr, Rosana Rodrigues de Sousa, Rumão Batista Nunes de Carvalho, Gilvo de Farias, Laura Maria Feitosa Formiga, Artemizia Francisca de Sousa, Maria Regina Alves Cardoso, Betzabeth Slater, Wolney Lisbôa Conde, Adriana de Azevedo Paiva, Karoline de Macêdo Gonçalves Frota

**Affiliations:** I Universidade Federal do Piauí Departamento de Nutrição Programa de Pós-Graduação em Alimentos e Nutrição Teresina PI Brasil Universidade Federal do Piauí. Campus Ministro Petrônio Portella. Departamento de Nutrição. Programa de Pós-Graduação em Alimentos e Nutrição. Teresina, PI, Brasil; II Universidade Federal do Piauí Picos PI Brasil Universidade Federal do Piauí. Campus Senador Helvídio Nunes de Barros. Curso de Graduação Bacharelado em Nutrição. Picos, PI, Brasil; III Universidade Federal do Piauí Picos PI Brasil Universidade Federal do Piauí. Campus Senador Helvídio Nunes de Barros. Curso de Graduação Bacharelado em Enfermagem. Picos, PI, Brasil; IV Universidade Federal do Piauí Departamento de Nutrição Teresina PI Brasil Universidade Federal do Piauí. Campus Ministro Petrônio Portella. Departamento de Nutrição. Teresina, PI, Brasil; V Universidade de São Paulo Faculdade de Saúde Pública Departamento de Epidemiologia São Paulo SP Brasil Universidade de São Paulo. Faculdade de Saúde Pública. Departamento de Epidemiologia. São Paulo, SP, Brasil; VI Universidade de São Paulo Faculdade de Saúde Pública Departamento de Nutrição São Paulo SP Brasil Universidade de São Paulo. Faculdade de Saúde Pública. Departamento de Nutrição. São Paulo, SP, Brasil

**Keywords:** Health Surveys, methods, Data Collection, methods, Cluster Sampling, Stratified Sampling

## Abstract

**OBJECTIVE:**

To describe the methodological aspects of the Piauí home healthcare survey (ISAD-PI) and assess the relation between sampling plan, precision, and design effects, assuming that population health surveys are relevant instruments for health monitoring.

**METHODS:**

ISAD-PI was a population-based cross-sectional study that analyzed the living conditions and health status of the population residing in urban areas in the municipalities of Teresina and Picos, in Piauí. Sampling was carried out by conglomerates in two stages: Primary Sampling Units and households. To calculate the sample size, we considered the stratification of the population in both cities, according to the age of the individuals. We evaluated the “non-response” rate (NRR) and estimated the proportions according to sex and age, as well as the prevalence of social determinants of health in order to assess the compliance of the sampling plan. Analyses related to the precision according to the coefficient of proportion variation of the standard error (*Cv-pˆ*) and the design effect (*deff*). *Cv-pˆ* less than 20% and *deff* less than 1.5 were considered adequate. The total NRR of households was 38.2% in Teresina and 38.3% in Picos. We carried out twenty-four proportion estimates in relation to age and sex and 48 prevalence of social and health determinants estimates, totaling 72 estimates. Among them, 71 had *Cv-pˆ* less than 20% and 61 had deff *less* than or equal to 1.5.

**CONCLUSION:**

Data generated from the ISAD-PI may contribute to the assessment of health and morbidity conditions in the population. Furthermore, methodological aspects employed in this research may serve as a basis for studies carried out in other cities in Brazil.

## INTRODUCTION

Population healthcare surveys are instruments of great importance for monitoring the health situation of the population, providing data that support the planning of action strategies, investments, and implementation of new health policies, as well as allowing the evaluation of health policies already existing^1,2^.

Epidemiological surveys cover a wide universe of individuals and, given the impossibility to consider the participation of the whole population in this research, we worked with representative samples. In this context, the sampling plan represents the set of techniques used to calculate the sample size and sample selection^3^.

Publications with greater detail on the sampling plans used in epidemiological research in the health area have intensified and might serve as a basis for new experiences. Besides, it could provide tools for the design of other studies^4^.

The cluster sampling plan with stratification has been used in health surveys with national coverage, such as the *Pesquisa Nacional de Saúde* (PNS - National Health Survey)^6^ and the *Pesquisa Nacional de Saúde Bucal*^5^ (National Oral Health Survey), and with municipal coverage, such as the *Inquérito de Saúde de São Paulo* (ISA -CAPITAL - São Paulo Health Survey)^4^. In these studies, the coefficient of variation (*Cv*) and the design effect (*deff*) have been used to assess the sampling plan^4^, stipulated from estimates of the prevalence of health determinants such as self-assessment of health, use of health services, and diagnosis of chronic diseases.

We considered the “*Inquérito de Saúde Domiciliar no Piauí* - ISAD-PI” (“Home Health Survey in Piauí - ISAD-PI”) to analyze determinants and health conditions of the population residing in urban areas in the municipalities of Teresina and Picos in Piauí. Thus this study aim to describe the methodological aspects of ISAD-PI as a population health survey and assess the compliance of the sampling plan in relation to precision and design effects.

## METHODS

Description of the ISAD-PI, with an emphasis on the study population, sampling, and collection procedures, in addition to the presentation of the results of the application of the sampling plan.

### Study Characterization and Research Subjects

The ISAD-PI was a population-based, cross-sectional study that intended to analyze determinants and health conditions of the population residing in urban areas of Teresina and Picos, in Piauí. For the study, all individuals residing in private households were eligible, except individuals with disabilities that hampered research.

Piauí is a state in the Northeast of the country located between 2º 44’ 49” and 10º 55’ 05” south latitude and between 40º 22’ 12” and 45º 59’ 42” west longitude. This Brazilian state limits with Ceará, Pernambuco, Bahia, Tocantins, and Maranhão. The state of Piauí has an area of 251,616,823km^2^ and has 224 municipalities^8^. The choice of the two municipalities included in the research took into account the four demographic mesoregions of Piauí: Northern, Center-North region, Southeast and Southwest regions, with Teresina being the state capital and the municipality with the highest population density in the Center-north region and Picos being the third-largest city in the state (located 308km from the capital Teresina), with the highest population density in southeastern Piauí. In addition, these municipalities have campuses of the Universidade Federal do Piauí (Federal University of Piauí), the institution responsible for data collection, making logistics feasible.

### Sample Size

To calculate the sample size, the size of the population of Teresina (767,557 inhabitants) and the population of Picos (58,321 inhabitants)^9^ were considered, as well as the stratification of the population of both cities, according to the age of the individuals to both sexes: children under 2 years old; children aged 3 to 4 years; children from 5 to 9 years old; adolescents aged 10 to 14 years; adolescents aged 15 to 19 years; adults aged 20 to 59 years, and older adults over 60 years (Table 1 - Block 1).

**Table 1:** Reference population, person/household ratio, planned sample of households and persons and simulation for 50% proportion estimates, according to age and sex groups in Teresina and Picos. PI, ISAD-PI, 2019.

City/Sex	Age (years)							

	0–2	3–4	5–9	10–14	15–19	20–59	≥ 60	
Block 1	Population reference							

Teresina								
M	16,652	11,373	29,010	33,404	33,807	206,062	26,299	
F	16,085	10,905	28,073	32,820	36,003	248,680	38,384	
Picos								
M	1,187	848	2,237	2,598	2,689	15,600	2,263	
F	1,258	820	2,201	2,522	2,824	18,144	3,130	

Block 2	Person/household ratio							

Teresina								
M	0.0793	0.0541	0.1381	0.1590	0.1609	0.9808	0.1252	
F	0.0766	0.0519	0.1336	0.1562	0.1714	1.1837	0.1827	
Picos								
M	0.0701	0.0500	0.1320	0.1533	0.1587	0.9207	0.1336	
F	0.0742	0.0484	0.1299	0.1488	0.1667	1.0708	0.1847	

Block 3	Sample of households to guarantee at least 30 individuals per group							

Teresina								
M	379	554	217	189	186	31	240	
F	392	578	225	192	175	25	164	
Picos								
M	428	599	227	196	189	33	225	
F	404	620	231	202	180	28	162	

Block 4	Sample size of individuals							

Teresina								Total
M	46	31	80	92	93	567	72	981
F	44	30	77	90	99	684	106	1,130
Total	90	61	157	182	192	1251	178	2,111
Picos								Total
M	43	31	82	95	98	571	83	1,003
F	46	30	81	92	103	664	115	1,131
Total	89	61	163	187	201	1235	198	2,134

Bloco 5	Simulation of the behavior of the confidence interval (95%CI) and the coefficient of variation of the standard error (*Cv*) for proportions (*pˆ*) 50%							

Teresina	95%CI (*Cv-pˆ*)							

M	35.5–64.5 (14.7)	32.4–67.6 (18.0)	39.0–61.0 (11.2)	39.8–60.2 (10.4)	39.8–60.2 (10.4)	45.8–54.2 (4.2)	38.5–61.6 (11.8)	
F	35.2–64.8 (15.1)	32.1–67.9 (18.26)	38.8–61.2 (11.4)	39.7–60.3 (10.5)	40.2–59.8 (10.1)	46.3–53.8 (3.8)	40.5–59.5 (9.7)	
Picos								
M	35.1–64.9 (15.3)	32.4–67.6 (18.0)	39.2–60.8 (11.0)	40.0–60.1 (10.3)	40.1–59.9 (10.1)	45.9–54.1 (4.2)	39.2–60.7 (11.0)	
F	35.6–64.5 (14.7)	32.1–67.9 (18.3)	39.1–60.9 (11.1)	39.8–60.3 (10.4)	40.3–59.7 (9.8)	46.2–53.8 (3.9)	40.8–59.1 (9.3)	

ISAD-PI: Home Health Survey in Piauí; M: male; F: female; 95%CI: 95% confidence interval; *Cv-pˆ*: coefficient of variation of the standard error of the proportion.

The municipality of Teresina had 210,093 private households in 2010 and that Picos had 16,944^9^. Therefore, we calculated the average number of individuals in each age group per household (Table 1 - Block 2).

The distribution of sample means can be approximated by a normal distribution if n > 30 and the population has any distribution^10^; so in order to ensure that a minimum of 30 individuals of each age group, for both sexes participated in the sample, we estimated the number of households needed for each age group (Table 1 - Block 3). Thus, the largest sample size of households relates to the female age group of 3-4 years, both in Teresina (n_0_ = 578 households) and in Picos (n_0_ = 620 households). The expected number of individuals for each age group and sex was obtained, presented in Table 1 - Block 4.

Considering the age ranges (Tabela 1 – Block 4), studies were carried out by simulating the 95% confidence interval (95%CI) and the coefficient of variation of the standard error of the proportion – *Cv-pˆ* for estimates of the proportion (*pˆ* ), ranging from 10% to 70%, according to age groups, sex, and respective sample sizes (values for estimates of 50% can be seen in Table 1- Block 5).

Taking into account that losses may occur during data collection due to various reasons, such as the absence of the resident in the selected household, refusal of the resident to answer the questionnaire, errors in the answers, or even closed household, the size of the final sample for this study was adjusted using n = n_0_/0,90, assuming a response rate of 90%, resulting in n ≅ 642 households in Teresina and n ≅ 688households in Picos.

### Sampling Plan

The study’s sampling plan was carried out by a cluster sampling process, in two stages: Primary Sampling Units (PSU) and households, based on census data from the Brazilian Institute of Geography and Statistics (IBGE) in 2010^9^.

The PSU are area units set apart to the sampling plans. The census sector, composed of approximately 300 families and approximately 1,000 inhabitants, is the smallest available geographic unit for which there is data on residents with similar socioeconomic characteristics.

In the first stage, we intended to improve sampling efficiency and generate similarly sized PSU (in relation to the number of households). So when necessary the census tracts of each city were divided or grouped in such a way that the coefficient of variation for their size did not exceed 10%. Thus, the generated PSU could be constituted by a single census tract, a fraction of a census tract, or a grouping of census tracts^11^.

Then we ordered the PSU according to their code, so all urban areas of the municipalities included in the research were represented in the sample. Thus, a systematic sample was taken from this ordered list of PSU in each city, with probability proportional to size. To facilitate the estimation of the parameters of interest, we defined that 30 PSU in Teresina and 24 PSU in Picos would be selected with equiprobability.

The second stage involved the systematic sampling of households within each UPA selected in the first stage. The fix sampling fraction in the second stage caused the number of selected households to be higher (or lower) than planned if the census sector had grown (or decreased) since the 2010 Census. Thus the second-stage sampling fraction can be rewritten by: $\frac{b(M_{i}^{\prime }/M_{i}}{M_{i}}$ , whereby M_i_’ is the number of households in the PSU “i” obtained in the household listing activity carried out in the field.

The global sampling fraction used in this study was: $f=\frac{{aM}_{i}}{\sum M_{i}}\times \frac{b}{M_{i}}$ where: f = global sampling ration; a = total number of PSU to be selected in the first stage; M_i_ = number of households in PSU “i*”*; b = number of households to be drawn in each selected UPA.

### Household Non-Response Rate

Cases in which the team made initial contact with the household were classified as “non-response” due to refusal of the residents (NR-Refusal) and, after clarification on the research procedures, the residents refused to participate in the study. The cases in which the household remained closed after three attempts were classified as “non-response” due to the absence of residents (NR-Absence) and the total number of households with impediment (NR-Total) corresponds to the sum of the two values.

“Non-Response” Rates (NRR) were calculated as follows: *NRR* – *Total* = *x*/*X*; NRR – Absence = xi/X and ; NRR – Absence = xz/X where: x = total number of households with “non-response”; xi = number of households with “non-response” due to refusal by residents; xz = number of households with “non-response” due to the absence of residents; and X = number of households in the sample.

### Data Collection Logistics

Data were collected between September 2018 and February 2020. During this period, with the help of IBGE’s digital grids^11^, the streets of the PSU were always covered in a clockwise direction, with the supervisor’s right arm facing the houses to count the households. When selected, a multi-professional trained team visited a household.

After clarification on ethical procedures and data collection, residents of the households were invited to participate in the research and, in case of acceptance and by signing the Informed Consent Form and the Free and Informed Assent (for minors). Data collection was started immediately or scheduled according to the availability of residents.

If any selected household was unavailable on the first visit, the researchers returned to the household three times, on different days and times, including one day on the weekend or on a holiday. After the attempts, if they remained closed, these households were excluded. In the case of closed households, the team used an informative pamphlet about the research, containing clarifications, a telephone number, and an invitation to participate.

To give visibility to the Survey and with a view to clarifying the population in general about the existence of the research, the study was disseminated in various media such as radio, social networks, newspapers broadcast on open TV, and written newspapers. Furthermore, information leaflets were distributed at the research sites.

The collection in the households was carried out in two stages. The first stage took place by the application of structured questionnaires by trained interviewers with mobile devices (cellphones and tablets) and the Epicollect *5®* (Imperial College London, 2018), available at https://five.epicollect.net/. The questionnaires used in the survey were based on questionnaires previously used in population-based surveys developed in the country: *Inquérito de Saúde no município de São Paulo* (Health Survey in the city of São Paulo), in 2015^12^, and *Pesquisa Nacional de Saúde* (National Health Survey), in 2013^13^.

Teams collected sociodemographic, economic, access to health and sanitation services, current and past health status, violence and safety, self-reported morbidities, medication use, lifestyle, eating habits, and physical activity data. Moreover, at this stage, anthropometric data were also collected (weight, height, waist circumference, arm circumference, calf circumference, and skin folds) and blood pressure was measured. In order to reduce errors, all anthropometrists and technicians responsible for measuring blood pressure were trained and standardized according to the same methodology, and all measurements were collected in duplicate. Specifically for anthropometric measurements, training was carried out by the team from the Laboratory of Nutritional Assessment of Populations (LANPOP), from the Faculdade de Saúde Pública of the Universidade de São Paulo (Faculty of Public Health/ University of São Paulo), a partner in carrying out the ISAD-PI.

Questions related to the household, including access to health and sanitation services, family income, and goods were addressed to the head of the family, identified by the residents themselves. In the absence of the head of household or his refusal to answer the questions, another capable family member answered the questions.

Due to logistical reasons, only in the municipality of Teresina we carried out a second collection stage, following the same sampling plan, so 50% of the households drawn in each sector were systematically selected, forming a sub-sample. For this, in addition to the data collected in the first stage, adolescents (10 to 19 years old), adults (20 to 59 years old), and older adults (60 years old or more) residing in households included in the sub-sample, households were invited to participate in data collection on food consumption, by the application of 24-hour food recall (Sample-R24hs) and blood collection (Blood-sample) for subsequent biochemical analysis (glycemia and lipid profile).

Blood collection was performed at home by trained nurses, on days scheduled according to the availability of residents. Guidance for participants was provided in writing at the first home visit and reinforced by phone call the day before the collection date. They followed a standardized script, including 12-hour fasting for food and non-alcoholic beverages, 72-hour fasting for alcoholic beverages, and no physical activity or physical effort on the day scheduled for collection.

The R24hs was applied by trained nutritionists, using the multiple-pass *method interview technique*, which consists of an interview guided in five stages with the objective of reducing underreporting of food consumption^14^. Furthermore, after an interval of 2 months, face-to-face reapplication of the 24-hour recall was performed in 40% of the sample that responded to the first recall.

### Evaluated Variables

In this study, to assess the compliance of the sampling plan in relation to precision and design effects, variables related to sociodemographic aspects, self-assessment in health, use of health services, and health assessment were used, usually studied in surveys of health^4^.

The variables were presented to the participants as follows: 1 - gender (What is your gender? male | female); 2 - age (How old are you?); 3 - skin color (What is your skin color? white | black | yellow | brown | indigenous | another | do not know); 4 - health self-assessment (In general, how do you rate your health? very good | good | regular | too bad | bad | do not know); 5 - use of family health services (Have you ever used the services offered by the *Programa Saúde da Família/Estratégia Saúde da Família* (PSF/ESF - Family Health Program/Family Health Strategy)? yes | no | do not know); 6 - health assessment (When was the last time you had your blood pressure measured? less than 6 months ago | between 6 months and less than 1 year | between 1 year and less than 2 years | between 2 years and less than 3 years | 3 years or more | never | do not know).

### Coefficient of Variation (Cv) and Design Effect (deff)

The parameters estimated in this study were the proportions according to sex and age, as well as the prevalence of people who declared themselves black or brown, reported very good or good self-rated health, had already used the family health program, and had their blood pressure measured in the last 6 months.

Estimates of proportion and prevalence were analyzed for accuracy using the coefficient of variation (*Cv*). Estimates with *Cv* less than 20% were considered accurate enough. In addition, we also evaluated the design effect measures (*deff*), used as efficiency measures of complex sampling designs^15^. Values less than 1.5 were considered adequate. The frequency of estimates smaller than two was also verified, which is a value frequently adopted in sampling plans in health surveys^4,7,5^.

For the variables that had missing values, it was decided to perform the imputation by the *Predictive Mean Matching* method, since the percentage of imputed data did not exceed 20%. This procedure kept the original equiprobabilistic design of the sample. All analyzes were carried out in the *survey* module of the *Stata* program version 14, which considers the sampling design in estimating the results.

## RESULTS

30 PSU were drawn in Teresina and 24 in Picos^16^ (Figure). In total, 1,285 people were interviewed in Teresina, corresponding to 42.8 (16.4) individuals per PSU, and 1013 people in Picos, which correspond to 42.2 (18.1) individuals per PSU. The survey ended with the final total sample of 497 households in Teresina and 441 in Picos (Table 2). The sub-samples Sample-R24h and Sample-blood comprised, respectively, 617 and 421 people in Teresina.

**Figure:**
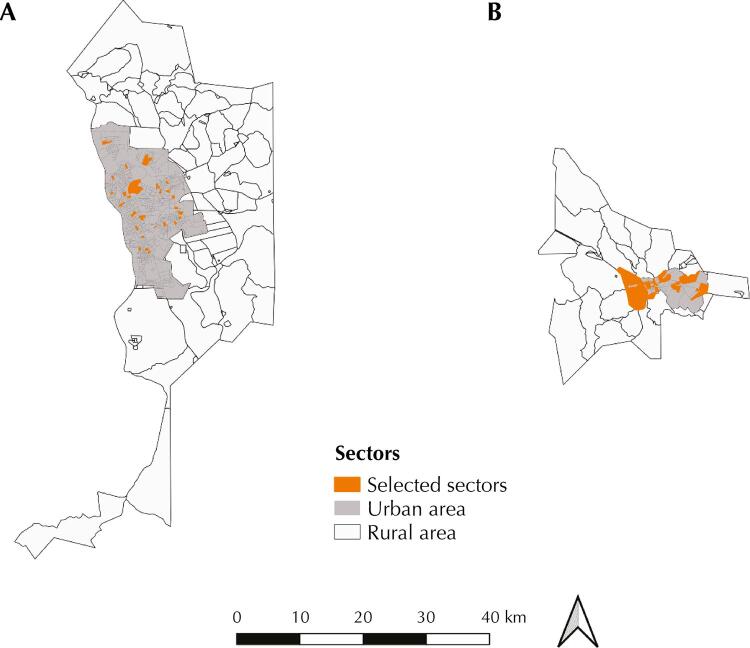
Census sectors in the municipalities of Teresina (A) and Picos (B), in Piauí, participating in ISAD-PI.

Source: Extracted from Silva (2020)^16^ - Prepared in the *software* QGIS version 3.10.8 *for Windows* from the digital grids made available by IBGE for the year 2010.

Note: Population-Based Health Survey in the Municipalities of Teresina and Picos – PI.

**Table 2:** Interviews carried out, households visited, and the “non-response” rate (NRR) of households according to the primary sampling unit (PSU) in Teresina and Picos. Piauí, ISAD-PI, 2019.

	Teresina					Picos					
	
	Interviews conducted	Drawn households	172 interviewed households.	% NRR Absence	% Total NRR	Interviews conducted	Drawn households	172 interviewed households.	% NRR (Refusal)	% NRR Absence	% Total NRR
	48	49	15	11.5	42.3	37	43	18	9.3	48.8	58.1
	42	12	19	9.4	40.6	35	33	17	9.1	39.4	48.5
	9	24	5	25.0	68.8	23	27	13	14.8	37.0	51.9
	17	13	7	5.9	35.3	27	28	14	7.1	42.9	50.0
	33	26	15	40.0	40.0	35	32	18	15.6	28.1	43.8
	39	33	15	26.9	42.3	54	38	22	10.5	31.6	42.1
	78	36	30	12.2	38.8	32	26	14	23.1	23.1	46.2
	33	51	12	0.0	0.0	72	37	33	5.4	5.4	10.8
	25	17	11	16.7	54.2	48	25	20	8.3	8.3	16.7
	30	20	13	0.0	0.0	33	26	17	7.7	26.9	34.6
	47	26	15	7.7	42.3	9	12	6	8.3	41.7	50.0
	40	29	18	15.2	45.5	40	30	17	6.7	36.7	43.3
	75	31	24	11.1	33.3	48	28	19	14.3	17.9	32.1
	60	32	25	15.7	51.0	25	10	10	0.0	0.0	0.0
	49	23	17	0.0	0.0	61	35	26	17.1	8.6	25.7
	17	28	11	35.0	45.0	35	34	16	14.7	38.2	52.9
	50	31	20	11.5	23.1	36	17	17	0.0	0.0	0.0
	38	30	15	37.9	48.3	27	32	11	31.3	34.4	65.6
	62	35	20	22.6	35.5	28	29	11	20.7	41.4	62.1
	61	31	20	22.6	38.7	39	30	18	13.8	24.1	37.9
	25	13	9	21.7	60.9	78	37	29	13.5	8.1	21.6
	46	28	15	17.9	46.4	65	43	24	14.0	30.2	44.2
	54	22	17	22.6	45.2	46	20	20	0.0	0.0	0.0
	29	26	16	33.3	46.7	80	45	31	15.6	15.6	31.1
	53	26.8	27	8.6	22.9	-	-	-	-	-	-
	45	49	17	16.1	45.2	-	-	-	-	-	-
	27	12	13	0.0	0.0	-	-	-	-	-	-
	54	24	19	17.9	32.1	-	-	-	-	-	-
	49	13	17	0.0	22.7	-	-	-	-	-	-
	50	26	20	3.8	23.1	-	-	-	-	-	-

Mean ± DP	42.8 ± 16.4	26.8 ± 9.3	19.0 ± 5.5			42.2 ± 18.1	29.9 ± 9	18.4 ± 6.6			

Total	1,285	804	497		38.2	1,013	717	441			38.3

ISAD-PI: Home Health Survey in Piauí; PSU: primary sampling unit; %RR: % response rate; %OF: % offside rate.

The numbers of households drawn in Teresina and Picos (804 and 715, respectively; Table 2) were higher than those considered necessary for conducting the interviews, provided for in the sampling stage (642 and 688, respectively). Despite this, the number of households interviewed was lower than planned, considering the average total “non-response” rate of 38.2% in Teresina and 38.3% in Picos, both higher than expected, of 10%.

Of the total number of households in which there was “non-response” in Teresina, 21.64% occurred due to residents’ refusal to participate in the survey and 16.54% occurred due to exclusion given the absence of residents after three attempts. In Picos, impediments due to refusal and absence of the resident in three attempts accounted for 12.45% and 25.87%, respectively.

We carried out twenty-four estimates of the proportion in relation to age and sex (Table 3) and 48 estimates of the prevalence of social and health indicators (Table 4), totaling 72 estimates. Except for the estimated proportion of male adolescents in the blood sample (*Cv-pˆ* = 27,6), the other estimates showed *Cv-pˆ* less than 20%.

**Table 3:** Interviews, proportion estimates, confidence intervals, coefficients of variation and design effects according to sex of adolescents, adults and older adults, in Teresina and Picos. Piauí, ISAD-PI, 2019.

	Male			Female		
	
	n	pˆ (95%CI)	Cv | deff	n	pˆ (95%CI)	Cv (deff)
**Teresina (Total sample)**						

Teens (10–19 years old)	92	21.6(16.0–28.6)	14.3 | 2.4)	135	19.3(16.0–23.0)	8.8 | 1.3
Adults (20–59 years old)	252	59.3(55.3–63.2)	3.3 | 0.7	413	59.0(56.5–61.4)	2.0 | 0.4
Older adults (60+ years old)	81	19.0(14.8–24.1)	12.0 | 1.4	152	21.7(17.7–26.4)	9.8 | 1.9

**Teresina (Blood sample)**						

Teens (10–19 years old)	32	22.7(12.3–38.0)	27.6 | 3.1	51	18.2(14.2–23.0)	11.7 | 0.8
Adults (20–59 years old)	75	53.2(45.8–60.4)	6.7 | 0.7	167	59.6(54.6–64.5)	4.0 | 0.7
Older adults (60+ years old)	34	24.1(16.1–34.4)	18.6 | 1.5	62	22.1(17.8–27.2)	10.2 | 0.8

**Teresina (R24h sample)**						

Teens (10–19 years old)	52	24.0(17.5–31.9)	14.7 | 1.5	68	17.0(14.2–20.2)	8.6 | 0.6
Adults (20–59 years old)	120	55.3(50.5–50.0)	4.2 | 0.5	245	61.2(57.2–65.1)	3.1 | 0.6
Older adults (60+ years old)	45	20.7(14.8–28.3)	15.8 | 1.4	87	21.8(17.4–26.9)	10.6 | 1.2

**Picos**						

Teens (10–19 years old)	75	22.1(18.2–26.5)	9.2 | 0.8	80	14.7(12.0–17.8)	9.5 | 0.8
Adults (20–59 years old)	215	63.2(58.4–67.8)	3.6 | 0.7	368	67.4(62.5–72.0)	3.4 | 1.3
Older adults (60+ years old)	50	14.7(11.0–19.4)	13.9 | 1.1	98	17.9(13.5–23.5)	13.5 | 2.1

ISAD-PI: Health Survey of Piauí; M: male; F: female; n: interviews carried out; *pˆ*: proportion; 95%CI: 95% confidence interval; *Cv*: coefficients of variation.

**Table 4:** Prevalence estimates, confidence intervals, coefficients of variation and design effects according to sex of adolescents, adults and older adults in Teresina and Picos. Piauí, ISAD-PI, 2019.

Indicators	Male		Female	
	
	pˆ (95%CI)	Cv(*deff*)	pˆ (95%CI)	Cv(*deff*)
**Teresina**				

Teens (10–19 years old)				
Very good or good health self-assessment	75.6 (65.5–83.4)	5.8 | 0.9	64.0 (55.1–71.8)	6.4 | 0.9
Have you already used the family health program	78.9 (67.4–87.1)	6.1 | 1.2	84.9 (77.5–90.3)	3.6 | 1.9
Blood pressure measured in the last 6 months	35.6 (26.6–46.6)	13.3 | 0.9	38.3 (28.7–48.9)	13.0 | 1.4
Self-declared black or brown	75.5 (64.5–84.0)	6.3 | 1.1	80.5 (74.2–85.5)	3.4 | 0.6
Adults (20–59 years old)				
Very good or good health self-assessment	64.5 (57.4–71.0)	5.2 | 1.2	55.5 (29.9–42.6)	4.0 | 0.8
Have you already used the family health program	64.5 (57.3–71.1)	5.2 | 1.2	79.6 (73.3–84.7)	3.5 | 1.9
Blood pressure measured in the last 6 months	62.0 (56.0–67.8)	4.7 | 0.9	66.7 (61.3–71.6)	3.8 | 1.2
Self-declared black or brown	79.2 (72.8–84.4)	3.6 | 1.2	77.4 (72.8–81.3)	2.7 | 0.9
Older adults (60+ years old)				
Very good or good health self-assessment	46.2 (35.8–56.8)	11.3 | 0.8	30.4 (22.6–39.6)	13.9 | 1.2
Have you already used the family health program	79.5 (62.5–90.0)	8.5 | 2.0	81.1 (71.8–87.8)	4.8 | 1.5
Blood pressure measured in the last 6 months	79.5 (70.3–86.4)	4.9 | 0.7	90.4 (84.8–94.2)	2.5 | 0.9
Self-declared black or brown	82.0 (69.0–90.0)	6.3 | 1.4	77.0 (67.9–84.2)	5.2 | 1.3

**Picos**				

Teens (10–19 years old)				
Very good or good health self-assessment	82.4 (70.7–90)	5.6 | 1.0	80.5 (67.2–89.3)	6.6 | 1.4
Have you already used the family health program	76.5 (67.9–83.3)	4.8 | 0.5	84.4 (75.2–90.6)	4.4 | 0.8
Blood pressure measured in the last 6 months	33.8 (22.6–47.3)	17.9 | 1.1	33.8 (22.4–44.6)	14.6 | 0.8
Self-declared black or brown	57.4 (44.6–69.2)	10.6 | 1.0	67.5 (54.1–78.6)	8.9 | 1.3
Adults (20–59 years old)				
Very good or good health self-assessment	67.3 (61.7–72.5)	3.9 | 0.6	55.1 (49.2–61.0)	5.2 | 1.2
Have you already used the family health program	60.5 (53.0–67.5)	5.9 | 1.1	87.2 (83.0–90.5)	2.0 | 1.0
Blood pressure measured in the last 6 months	56.1 (50.2–61.8)	5.0 | 0.7	70.7 (63.577.1)	4.7 | 1.8
Self-declared black or brown	69.3(60.2–77.1)	6.0 | 1.6	71.6 (64.0–78.1)	4.8 | 2.0
Older adults (60+ years old)				
Very good or good health self-assessment	47.9(33.7–62.5)	14.9 | 1.0	44.8 (33.1–57.0)	13.2 | 1.3
Have you already used the family health program	81.3(70.1–90.0)	5.6 | 0.6	89.6 (74.1–96.3)	5.5 | 2.5
Blood pressure measured in the last 6 months	80.9(69.1–88.9)	5.9 | 0.7	81.1 (71.2–88.1)	5.0 | 1.0
Self-declared black or brown	66.7(50.8–79.5)	10.6 | 1.1	65.6 (54.9–75.0)	7.5 | 1.0

ISAD-PI: Home Health Survey in Piauí; M: male; F: female; *pˆ*: proportion; 95%CI: 95% confidence interval; Cv: standard error coefficient of variation; *deff*: design effect.

The effect of the proportion design in relation to sex and age (Table 3) was less than 1.5 for 20 of the 24 estimates. The proportion estimates that presented *deff* greater than 1.5 were the proportion of elderly females in Teresina (*deff* = 1.9) and in Picos (*deff =* 2.1), as well as the proportion of male adolescents in Teresina (*deff* = 2.4) and proportion of male adolescents in the blood sample.

Regarding the prevalence estimates of social and health indicators (Table 4), the design effect was less than 1.5 in 87.5% of the estimates (42 of the 48 estimates) and less than 2 in 95.8% of the estimates (46 out of 48 estimates).

## DISCUSSION

The ISAD-PI stands out for being a household population study that addresses health determinants and conditions in two cities in Piauí and encompasses various health components. It is expected to obtain more specific and detailed health data and indicators, meeting the needs at the municipal and regional level, and contributing with relevant information for national surveys.

Brazil is a large country, with great socioeconomic diversity^9^, so it is essential to carry out municipal surveys such as the ISAD-PI. Piauí has an HDI (Human Development Index) of 0.646 and is one of the Brazilian states with the lowest index, only higher than those of Maranhão (0.639) and Alagoas (0.631)^8^. Thus, cities in Piauí have a different reality from many cities in Brazil. Furthermore, the decision to include in the study the capital, Teresina-PI (IDH: 0.751) and a city in the interior of the state, Picos-PI (IDH: 0.698), favors the assessment of different realities within the state.

Regarding data collection procedures, the ISAD-PI took 18 months to complete, which can be considered a disadvantage in relation to the research costs. We emphasize the collection of detailed information on health and lifestyle, as well as the diversity of anthropometric measurements performed on all household members, at the expense of time and cost reduction. Anyway, this allowed for a deepening of the health information generated.

The number of households drawn was greater than that conceived in the sampling stage, mainly in the municipality of Teresina, a fact related to the increase in the number of households in the census sectors of Teresina and Picos since the 2010 census. The largest number of sampled households is the result of the application of constant fractions in the household drawing stage; thus, the equiprobability of the sample was maintained, which was provided by the drawing with probability proportional to size. However, the control over its final size was harmed.

Residents’ participation was lower than expected, despite the use of strategies such as dissemination of the research in the media, use of informative pamphlets, and the team returning to their homes more than once. Many refusals occurred because residents feared to open their homes or provide information to participate in the study. The “non-response” rate verified in this study can be used as a parameter for future research in the sample planning stage.

It is noteworthy that, although the number of households and, therefore, of participants, was lower than expected, the number of individuals in the final sample was considered appropriate for studies in public health, allowing adequate precision, in general with *Cv-pˆ* less than 20%. When stratification is used properly in the sampling plan, precision gains are obtained in the average estimates^17^. The decision to use stratification by sex and age group in the sampling stage of this study was satisfactory.

Cluster sampling designs, such as the one used in this study, present a risk of inaccuracy in estimated parameters. The *deff* measures the relationship between the estimators calculated according to the proposed sampling plan and a simple casual sample. This is a statistic commonly used to assess this effect^18,19^. In this study, *deff* values lower than 2 suggest that the difference between the values obtained according to the sample design used and a simple casual sample are not expressively different, indicating that the sample design was successful.

The ISAD-PI sample generated estimates close to those predicted, considering that most estimates presented *Cv* below 20% and that, in more than 90% of them, the *deff* was less than 1.5. Other epidemiological studies in the health area have proposed this value as an acceptable limit^3,4,7^. Furthermore, the accuracy verified in the study shows that the choice to maintain the self-weighting of the sample was correct, considering that the draw was equiprobabilistic.

The precision and effect of the design, examined in the evaluation of the estimates in this study, indicate that the data generated from the ISAD-PI may contribute to the assessment of health and morbidity conditions in the population, as well as to the assessment and implementation of health services and actions in the evaluated municipalities. Furthermore, methodological aspects employed in this research may serve as a basis for studies carried out in other cities in Brazil.

However, it is important to be aware of the use of results related to rare events, which must be interpreted with caution, in particular when performed with small samples. The few estimates with *deff* greater than 1.5 and *Cv* greater than 20% occurred mainly among adolescents and older adults, whose sample size was smaller than the adult stratum.

## FINAL CONSIDERATIONS

Representative health surveys are important for the assessment of health conditions in Brazilian municipalities, especially in cities in the northeast region. This region of Brazil lacks epidemiological studies with robust sampling methods that guarantee the representativeness of data. Moreover, it is important to highlight that their lifestyle and eating habits have particularities in relation to those practiced by the inhabitants of the South and Southeast regions, where most of the health surveys are carried out.

This survey was designed using a robust sampling technique, ensuring the representativeness of the data. Finally, household surveys present numerous challenges to researchers. For further development, subsidies are necessary and important to improve sampling designs and to discuss strategies of improvement.
