# Errata

**DOI:** 10.11606/s1518-8787.2022056003441err

**Published:** 2022-11-03

In the article “**Sampling plan and methodological aspects: a household healthcare survey in Piauí**”, DOI https://doi.org/10.11606/s1518-8787.2021055003441, published on the Revista de Saúde Pública.2021;55:118, on page 7, in the Table 2, the RSP corrects the header of third and eighth column.

Where it reads:

172 interviewed households.

It should read:

Interviewed households

