# Supplementary material for: Sampling plan and methodological aspects: a household healthcare survey in Piauí
Source: Rev Saude Publica. 2021 Dec 9;55:118. doi: 10.11606/s1518-8787.2021055003441 (PMC8664053; doi:10.11606/s1518-8787.2021055003441)
Supplement: Translation

# Translation: Plano de amostragem e aspectos metodológicos: inquérito de saúde domiciliar no Piauí

**Authors:** Lays Arnaud Rosal Lopes Rodrigues, Danilla Michelle Costa e Silva, Edina Araújo Rodrigues Oliveira, Layanne Cristina de Carvalho Lavôr, Rosana Rodrigues de Sousa, Rumão Batista Nunes de Carvalho, Gilvo de Farias, Laura Maria Feitosa Formiga, Artemizia Francisca de Sousa, Maria Regina Alves Cardoso, Betzabeth Slater, Wolney Lisbôa Conde, Adriana de Azevedo Paiva, Karoline de Macêdo Gonçalves Frota

**Affiliations:** I Brasil Universidade Federal do Piauí. Campus Ministro Petrônio Portella. Departamento de Nutrição. Programa de Pós-Graduação em Alimentos e Nutrição. Teresina, PI, Brasil; II Brasil Universidade Federal do Piauí. Campus Senador Helvídio Nunes de Barros. Curso de Graduação Bacharelado em Nutrição. Picos, PI, Brasil; III Brasil Universidade Federal do Piauí. Campus Senador Helvídio Nunes de Barros. Curso de Graduação Bacharelado em Enfermagem. Picos, PI, Brasil; IV Brasil Universidade Federal do Piauí. Campus Ministro Petrônio Portella. Departamento de Nutrição. Teresina, PI, Brasil; V Brasil Universidade de São Paulo. Faculdade de Saúde Pública. Departamento de Epidemiologia. São Paulo, SP, Brasil; VI Brasil Universidade de São Paulo. Faculdade de Saúde Pública. Departamento de Nutrição. São Paulo, SP, Brasil

**Keywords:** Inquéritos Epidemiológicos, métodos, Coleta de Dados, métodos, Amostragem por Conglomerados, Amostragem Estratificada

## Abstract

**OBJETIVO:**

Descrever os aspectos metodológicos do Inquérito de Saúde Domiciliar no Piauí (ISAD-PI), bem como avaliar a conformidade do plano amostral em relação à precisão e aos efeitos do desenho, dado que os inquéritos populacionais de saúde constituem instrumentos importantes para o monitoramento da situação de saúde da população.

**MÉTODOS:**

O ISAD-PI foi um estudo de base populacional, transversal, que analisou as condições de vida e situação de saúde da população residente nas áreas urbanas dos municípios de Teresina e Picos, no Piauí. A amostragem foi realizada por conglomerados, em dois estágios: Unidades Primárias de Amostragem e domicílios. Para o cálculo do tamanho da amostra, considerou-se a estratificação da população de ambas as cidades, de acordo com a idade dos indivíduos, para ambos os sexos. Para avaliação da conformidade do plano amostral, foi avaliada a taxa de “não-resposta” (TNR) e, além disso, as estimativas de proporções segundo sexo e idade, bem como as prevalências de determinantes sociais de saúde, que foram analisadas em relação à precisão por meio do coeficiente de variação da proporção do erro padrão (*Cv-pˆ*) e do efeito do delineamento (*deff*). Foram considerados adequados *Cv-pˆ* menores que 20%, e *deff* menores que 1,5. A TNR-Total dos domicílios foi de 38,2% em Teresina e de 38,3% em Picos. Foram realizadas 24 estimativas de proporção em relação à idade e ao sexo e 48 estimativas de prevalência de determinantes sociais e de saúde, totalizando 72 estimativas, das quais 71 apresentaram *Cv-pˆ* menor que 20% e 61 apresentaram *deff* menor ou igual a 1,5.

**CONCLUSÃO:**

Dados gerados a partir do ISAD-PI poderão contribuir para a avaliação das condições de saúde e morbidade na população. Ademais, aspectos metodológicos empregados nesta pesquisa poderão servir de base para estudos realizados em outras cidades do Brasil.

## INTRODUÇÃO

Os inquéritos populacionais de saúde constituem instrumentos de grande importância para o monitoramento da situação de saúde da população, fornecendo dados que permitem subsidiar o planejamento de estratégias de ação, investimentos e implantação de novas políticas de saúde, bem como permitem a avaliação das políticas de saúde já existentes^1,2^.

Pesquisas epidemiológicas abrangem um amplo universo de indivíduos e, diante da inviabilidade de se considerar a participação de toda população de interesse na pesquisa, busca-se trabalhar com amostras representativas. Neste contexto, o plano amostral representa o conjunto de técnicas utilizadas para cálculo do tamanho amostral e seleção da amostra^3^.

Atualmente, as publicações com maior detalhamento sobre os planos de amostragem utilizados em pesquisas epidemiológicas na área da saúde têm se intensificado e podem servir de base para novas experiências, além de fornecerem ferramentas para o delineamento de outros estudos^4^.

O plano amostral por conglomerados com estratificação tem sido empregado em pesquisas de saúde com abrangência nacional, como a Pesquisa Nacional de Saúde (PNS)^6^ e a Pesquisa Nacional de Saúde Bucal^5^, e com abrangência municipal, como o Inquérito de Saúde de São Paulo (ISA-CAPITAL)^4^. Nessas pesquisas, o coeficiente de variação (*Cv*) e o de efeito do delineamento (*deff*) têm sido empregados para a avaliação do plano amostral^4^, estipulados a partir de estimativas das prevalências de determinantes de saúde como autoavaliação de saúde, utilização de serviços de saúde e diagnóstico de doenças crônicas.

Com a finalidade de analisar determinantes e condições de saúde da população residente nas áreas urbanas dos municípios de Teresina e Picos no Piauí, desenhou-se o “Inquérito de Saúde Domiciliar no Piauí - ISAD-PI”. Portanto, o ISAD-PI é um inquérito populacional de saúde e o objetivo deste trabalho foi descrever seus aspectos metodológicos, bem como avaliar a conformidade do plano amostral em relação à precisão e aos efeitos do desenho.

## MÉTODOS

Descrição do ISAD-PI, com ênfase em população do estudo, procedimentos de amostragem e de coleta, além da apresentação dos resultados da aplicação do plano de amostragem.

### Caracterização do Estudo e Sujeitos da Pesquisa

O ISAD-PI foi um estudo de base populacional, transversal, que objetivou analisar determinantes e condições de saúde da população residente nas áreas urbanas dos municípios de Teresina e Picos, no Piauí. Para o estudo, eram elegíveis todos os indivíduos residentes em domicílios particulares, exceto aqueles que apresentassem quaisquer deficiências ou incapacidades que impossibilitassem a realização da pesquisa.

O Piauí é um estado do Nordeste do país situado entre 2º 44’ 49” e 10º 55’ 05” de latitude sul e entre 40º 22’ 12” e 45º 59’ 42” de longitude oeste, cujos limites são feitos com Ceará, Pernambuco, Bahia, Tocantins e Maranhão. O estado do Piauí possui área de 251.616,823km^2^ e conta com 224 municípios^8^. A escolha dos dois municípios incluídos na pesquisa levou em consideração as quatro mesorregiões demográficas do Piauí: Norte, Centro-norte, Sudeste e Sudoeste, sendo Teresina a capital do estado e município com maior densidade populacional do Centro-norte e Picos, a terceira maior cidade do estado (localizada a 308km da capital Teresina) e município com maior densidade populacional do sudeste piauiense. Além disso, os referidos municípios possuem campus da Universidade Federal do Piauí, instituição responsável pela coleta de dados, o que viabilizou a logística.

### Tamanho da Amostra

Para o cálculo do tamanho da amostra, considerou-se o tamanho da população de Teresina (767.557 habitantes) e da população de Picos (58.321 habitantes)^9^, assim como a estratificação da população de ambas as cidades, de acordo com a idade dos indivíduos para ambos os sexos: crianças menores de 2 anos; crianças de 3 a 4 anos; crianças de 5 a 9 anos; adolescentes de 10 a 14 anos; adolescentes de 15 a 19 anos; adultos de 20 a 59 anos e idosos com mais de 60 anos (Tabela 1 - Bloco 1).

**Tabela 1:** População de referência, razão pessoa/domicílio, amostra planejada de domicílios e pessoas e simulação para estimativas de proporção 50%, segundo grupos de idade e sexo em Teresina e em Picos. PI, ISAD-PI, 2019.

Cidade/Sexo	Idade (anos)							

	0–2	3–4	5–9	10–14	15–19	20–59	≥ 60	
Bloco 1	População de Referência							

Teresina								
M	16.652	11.373	29.010	33.404	33.807	206.062	26.299	
F	16.085	10.905	28.073	32.820	36.003	248.680	38.384	
Picos								
M	1.187	848	2.237	2.598	2.689	15.600	2.263	
F	1.258	820	2.201	2.522	2.824	18.144	3.130	

Bloco 2	Razão pessoa/domicílio							

Teresina								
M	0,0793	0,0541	0,1381	0,1590	0,1609	0,9808	0,1252	
F	0,0766	0,0519	0,1336	0,1562	0,1714	1,1837	0,1827	
Picos								
M	0,0701	0,0500	0,1320	0,1533	0,1587	0,9207	0,1336	
F	0,0742	0,0484	0,1299	0,1488	0,1667	1,0708	0,1847	

Bloco 3	Amostra de domicílios para garantir, no mínimo, 30 indivíduos por grupo							

Teresina								
M	379	554	217	189	186	31	240	
F	392	578	225	192	175	25	164	
Picos								
M	428	599	227	196	189	33	225	
F	404	620	231	202	180	28	162	

Bloco 4	Tamanho da amostra de indivíduos							

Teresina								Total
M	46	31	80	92	93	567	72	981
F	44	30	77	90	99	684	106	1.130
Total	90	61	157	182	192	1251	178	2.111
Picos								Total
M	43	31	82	95	98	571	83	1.003
F	46	30	81	92	103	664	115	1.131
Total	89	61	163	187	201	1.235	198	2.134

Bloco 5	Simulação do comportamento do intervalo de confiança (IC95%) e do coeficiente de variação do erro-padrão (*Cv*) para proporções (*pˆ*) de 50%							

Teresina	IC95% (*Cv-pˆ*)							

M	35,5–64,5 (14,7)	32,4–67,6 (18,0)	39,0–61,0 (11,2)	39,8–60,2 (10,4)	39,8–60,2 (10,4)	45,8–54,2 (4,2)	38,5–61,6 (11,8)	
F	35,2–64,8 (15,1)	32,1–67,9 (18,26)	38,8–61,2 (11,4)	39,7–60,3 (10,5)	40,2–59,8 (10,1)	46,3–53,8 (3,8)	40,5–59,5 (9,7)	
Picos								
M	35,1–64,9 (15,3)	32,4–67,6 (18,0)	39,2–60,8 (11,0)	40,0–60,1 (10,3)	40,1–59,9 (10,1)	45,9–54,1 (4,2)	39,2–60,7 (11,0)	
F	35,6–64,5 (14,7)	32,1–67,9 (18,3)	39,1–60,9 (11,1)	39,8–60,3 (10,4)	40,3–59,7 (9,8)	46,2–53,8 (3,9)	40,8–59,1 (9,3)	

ISAD-PI: Inquérito de Saúde Domiciliar no Piauí; M: masculino; F: feminino; IC95%: intervalo de confiança de 95%; *Cv-pˆ*: coeficiente de variação do erro-padrão da proporção.

Tendo em vista que o município de Teresina contava com 210.093 domicílios particulares, no ano de 2010, e que Picos contava com 16.944^9^, foi calculado o número médio de indivíduos de cada grupo etário por domicílio em cada cidade (Tabela 1 - Bloco 2).

Visto que a distribuição das médias amostrais pode ser aproximada por uma distribuição normal se o n > 30 e a população tem qualquer distribuição^10^, com o intuito de garantir que o mínimo de 30 indivíduos de cada grupo etário, para ambos os sexos, participassem da amostra, foi calculado o número de domicílios necessários para cada grupo etário (Tabela 1 - Bloco 3). Dessa forma, o maior tamanho de amostra em número de domicílios foi para o grupo etário de 3 a 4 anos do sexo feminino, tanto em Teresina (n_0_ = 578 domicílios) quanto em Picos (n_0_ = 620 domicílios). A partir disso, foi obtido o número esperado de indivíduos para cada grupo etário e sexo, apresentado na Tabela 1 - Bloco 4.

Considerando o número de indivíduos de cada faixa de idade (Tabela 1 – Bloco 4), foram realizados estudos por simulação do Intervalo de confiança de 95% (IC95%) e do coeficiente de variação do erro-padrão da proporção – *Cv-pˆ* para estimativas da proporção (*pˆ* ) variando de 10% a 70%, segundo grupos etários, sexo e respectivos tamanhos amostrais (valores para estimativas de 50% podem ser observados na Tabela 1- Bloco 5).

Levando em consideração que durante o levantamento de dados podem ocorrer perdas devido a vários motivos, como ausência do morador no domicílio sorteado, recusa do morador em responder ao questionário, erros nas respostas, ou mesmo domicílio fechado, o tamanho da amostra final para este estudo foi ajustado utilizando n = n_0_/0,90, admitindo-se uma taxa de resposta de 90%, resultando em n ≅ 642 domicílios em Teresina e n ≅ 688 domicílios em Picos.

### Plano de Amostragem

O plano de amostragem do estudo foi realizado por processo de amostragem por conglomerados, em dois estágios: Unidades Primárias de Amostragem (UPA) e domicílios, com base nos dados do censo do Instituto Brasileiro de Geografia e Estatística (IBGE) para o ano de 2010^9^.

As UPA constituem as unidades de área selecionadas para a amostra nos planejamentos amostrais. Já o setor censitário, composto por aproximadamente 300 famílias e aproximadamente 1.000 habitantes, é a menor unidade geográfica disponível para a qual existem dados dos residentes com características socioeconômicas semelhantes.

No primeiro estágio, para melhorar a eficiência da amostragem e gerar UPA com tamanho semelhante (em relação ao número de domicílios), os setores censitários de cada cidade foram, quando necessário, divididos ou agrupados de tal forma que o coeficiente de variação para o seu tamanho não excedesse 10%. Dessa forma, as UPA geradas poderiam ser constituídas por um único setor censitário, uma fração de um setor censitário, ou um agrupamento de setores censitários^11^.

As UPA foram, então, ordenadas de acordo com seu código, de forma que todas as áreas da zona urbana dos municípios incluídos na pesquisa estivessem representadas na amostra. Assim, uma amostra sistemática foi tomada a partir dessa lista ordenada das UPA de cada cidade, com probabilidade proporcional ao tamanho. Almejando facilitar a estimação dos parâmetros de interesse, foi definido que seriam selecionadas com equiprobabilidade 30 UPA em Teresina e 24 UPA em Picos.

O segundo estágio envolveu a amostragem sistemática de domicílios dentro de cada UPA selecionada no primeiro estágio. A fração de amostragem no segundo estágio foi fixada, fazendo com que o número de domicílios sorteados fosse maior (ou menor) do que o planejado, caso o setor censitário tivesse crescido (ou diminuído) desde o Censo 2010. Com essa opção, a fração de amostragem de segundo estágio pode ser reescrita por: $\frac{b\left(M_{i}^{\prime }/M_{i}\right)}{M_{i}^{\prime }}$ , em que M_i_’ é o número de domicílios na UPA “i” obtido na atividade de arrolamento de domicílios, realizada em campo.

A fração global de amostragem usada neste estudo foi: $f=\frac{{aM}_{i}}{\sum M_{i}}\times \frac{b}{M_{i}}$ onde: f = ração global de amostragem; a=número total de UPAs a serem selecionadas no primeiro estágio; M_i_ = número de domicílios na UPA “i*”*; b = número de domicílios a serem sorteados em cada UPA selecionada.

### Taxa de Não-Resposta de Domicílios

Foram classificados como “não-resposta” por motivo de recusa dos moradores (NR-Recusa) os casos em que a equipe conseguiu contato inicial com o domicílio e, após os esclarecimentos sobre os procedimentos da pesquisa, os moradores recusaram-se a participar do estudo. Já os casos em que o domicílio permaneceu fechado após três tentativas foram classificados como “não-resposta” por motivo de ausência dos moradores (NR-Ausência), e o número total de domicílios com impedimento (NR-Total) corresponde ao somatório dos dois valores.

As Taxas de “não-resposta” (TNR) foram calculadas da seguinte maneira: *TNR* – *Total* = *x*/*X*; TNR – Recusa = xi/X; e TNR – Ausência = xz/X; onde: x = número total de domicílios com “não-resposta”; xi = número de domicílios com “não-resposta” por recusa dos moradores; xz = número de domicílios com “não-resposta” por ausência de moradores; e X = número de domicílios da amostra.

### Logística de Coleta de Dados

Os dados foram coletados entre setembro de 2018 e fevereiro de 2020. Nesse período, com auxílio das malhas digitais do IBGE^11^, as ruas das UPA foram percorridas sempre no sentido horário, com o braço direito do supervisor voltado para as casas, fazendo a contagem dos domicílio, os quais, quando sorteados, foram visitados por uma equipe multiprofissional devidamente treinada.

Após os esclarecimentos sobre os procedimentos éticos e de coleta de dados, os moradores dos domicílios eram convidados a participar da pesquisa e, em caso de aceite e mediante assinatura dos Termos de Consentimento Livre e Esclarecido e Assentimento Livre e Esclarecido (para menores de idade), a coleta de dados era iniciada imediatamente ou agendada de acordo com a disponibilidade dos moradores.

Caso algum domicílio sorteado fosse encontrado fechado na primeira visita, os pesquisadores retornavam três vezes ao domicílio, em dias e horários diferentes, incluindo um dia em final de semana ou feriado. Após as tentativas, caso permanecessem fechados, esses domicílios eram excluídos. Um panfleto informativo sobre a pesquisa, contendo esclarecimentos, um número de telefone e um convite para participação, também foi utilizado como estratégia de contato com os moradores de domicílios fechados.

Para dar visibilidade ao Inquérito e com vistas a esclarecer a população de forma geral sobre a existência da pesquisa, o estudo foi divulgado em diversos meios de comunicação como rádio, redes sociais, jornais transmitidos em TV aberta e jornal escrito, além disso, panfletos informativos foram distribuídos nos locais da pesquisa.

A coleta nos domicílios foi realizada em duas etapas. A primeira etapa ocorreu mediante a aplicação de questionários estruturados por entrevistadores treinados, por meio de dispositivos móveis (celulares e *tablets*) e do aplicativo *Epicollect 5*^®^ (*Imperial College London, 2018*), disponível em https://five.epicollect.net/. Os questionários utilizados na pesquisa foram elaborados com base em questionários utilizados anteriormente em inquéritos de base populacional desenvolvidos no país: Inquérito de Saúde no município de São Paulo, em 2015^12^, e Pesquisa Nacional de Saúde, em 2013^13^.

Foram coletados dados sociodemográficos, econômicos, de acesso aos serviços de saúde e saneamento básico, estado de saúde atual e passado, violência e segurança, morbidades autorreferidas, uso de medicamentos, estilo de vida, hábitos alimentares e prática de atividade física. Além disso, nessa etapa, também foram coletados dados antropométricos (peso, altura, circunferência da cintura, circunferência do braço, circunferência da panturrilha e pregas cutâneas) e foi aferida a pressão arterial. De forma a reduzir erros, todos os antropometristas e técnicos responsáveis pela aferição da pressão arterial foram treinados e padronizados segundo uma mesma metodologia, e todas as medidas foram coletadas em duplicata. Especificamente para as medidas antropométricas, o treinamento foi realizado pela equipe do Laboratório de Avaliação Nutricional de Populações (LANPOP), da Faculdade de Saúde Pública da Universidade de São Paulo, parceira na realização do ISAD-PI.

Questões referentes ao domicílio, incluindo acesso aos serviços de saúde e saneamento, renda familiar e bens, eram direcionadas ao chefe da família, identificado pelos próprios moradores. Na ausência do chefe da família ou sua recusa para responder às questões, outro membro da família capacitado respondia às perguntas.

Por questões de logística, apenas no município de Teresina foi realizada uma segunda etapa de coleta, seguindo o mesmo plano de amostragem, de forma que 50% dos domicílios sorteados em cada setor foram sistematicamente selecionados formando uma subamostra. Para isso, além dos dados coletados na primeira etapa, adolescentes (10 a 19 anos), adultos (20 a 59 anos) e idosos (60 anos ou mais) residentes em domicílios incluídos na subamostra, eram convidados a participar da coleta de dados sobre consumo alimentar, por meio da aplicação de recordatório alimentar de 24 horas (Amostra-R24hs) e da colheita de sangue (Amostra-sangue) para posteriores análises bioquímicas (glicemia e perfil lipídico).

A colheita de sangue foi realizada no próprio domicílio por enfermeiros treinados, em dias agendados de acordo com a disponibilidade dos moradores. As orientações para os participantes foram fornecidas por escrito na primeira visita domiciliar e reforçadas por telefonema um dia antes da data da colheita. Elas seguiram um roteiro padronizado, incluindo: jejum de 12 horas de alimentos e bebidas não alcoólicas, jejum de 72 horas de bebidas alcoólicas e não realização de atividades físicas ou esforços físicos no dia agendado para a coleta.

O R24hs foi aplicado por nutricionistas treinados, com utilização da técnica de entrevista *multiple-pass method*, que consiste em uma entrevista orientada em cinco etapas, com o objetivo de reduzir o sub-relato do consumo alimentar^14^. Ademais, após um intervalo de 2 meses, foi realizada a reaplicação presencial do recordatório de 24 horas em 40% da amostra que respondeu ao primeiro recordatório.

### Variáveis Avaliadas

Neste estudo, para avaliação da conformidade do plano amostral em relação à precisão e aos efeitos do desenho, foram utilizadas variáveis relativas a aspectos sociodemográficos, à autoavaliação em saúde, ao uso de serviços de saúde e à avaliação de saúde, usualmente estudadas em inquéritos de saúde^4^.

As variáveis foram apresentadas aos participantes da seguinte forma: 1 - sexo (Qual o seu sexo ? masculino | feminino); 2 - idade (Qual a sua idade?); 3 - cor de pele (Qual é a sua cor da pele? branca | preta | amarela | parda | indígena | outra | não sabe); 4 - autoavaliação de saúde (Em geral, como o(a) sr.(a) avalia a sua saúde? muito boa | boa | regular | muito ruim | ruim | não sabe); 5 - utilização dos serviços de saúde da família (O(a) sr.(a) já utilizou os serviços oferecidos pelo Programa Saúde da Família/Estratégia Saúde da Família (PSF/ESF)? sim | não | não sabe); 6 - avaliação de saúde (Quando foi a última vez que o(a) sr.(a) teve sua pressão arterial medida? há menos de 6 meses | entre 6 meses e menos de 1 ano | entre 1 ano e menos de 2 anos | entre 2 anos e menos de 3 anos | 3 anos ou mais | nunca | não sabe).

### Coeficiente de Variação (Cv) e Efeito do Delineamento (deff)

Os parâmetros estimados neste estudo foram as proporções segundo sexo e idade, bem como as prevalências das pessoas que se autodeclararam pretas ou pardas, referiram autoavaliação de saúde muito boa ou boa, já tinham utilizado o programa de saúde da família e tinham aferido a pressão arterial nos últimos 6 meses.

As estimativas de proporção e prevalência foram analisadas quanto à precisão através do coeficiente de variação (*Cv*). As estimativas com *Cv* menor que 20% foram consideradas precisas o suficiente. Além disso, também foram avaliadas as medidas de efeito de delineamento (*deff*), utilizadas como medidas de eficiência de delineamentos complexos de amostragem^15^. Valores menores que 1,5 foram considerados adequados. A frequência de estimativas menores que dois também foi verificada, valor adotado, com frequência, em planos de amostragem em inquéritos de saúde^4,7,5^.

Para as variáveis que apresentavam valores faltantes, optou-se por realizar a imputação pelo método *Predictive Mean Matching*, sendo que o percentual de dados imputados não excedeu 20%. Esse procedimento manteve o desenho original, equiprobabilístico, da amostra. Todas as análises foram feitas no módulo *survey* do programa *Stata* versão 14, que considera o desenho amostral na estimativa dos resultados.

## RESULTADOS

Foram sorteadas 30 UPA em Teresina e 24 em Picos^16^ (Figura) e, no total, foram entrevistadas 1.285 pessoas em Teresina, que correspondem a 42,8 (16,4) indivíduos por UPA; e 1013 pessoas em Picos, que correspondem a 42,2 (18,1) indivíduos por UPA. O inquérito foi encerrado com a amostra final total de 497 domicílios em Teresina e 441 em Picos (Tabela 2). As subamostras Amostra-R24h e Amostra-sangue, em Teresina, compreenderam, respectivamente, 617 e 421 pessoas.

**Figura:** Setores censitários dos municípios de Teresina (A) e Picos (B), no Piauí, participantes do ISAD-PI.

Fonte: Extraído de Silva (2020)^16^ - Elaborado no *software* QGIS versão 3.10.8 *for Windows* a partir das malhas digitais disponibilizadas pelo IBGE para o ano de 2010.

Nota: Inquérito de Saúde de Base Populacional nos Municípios de Teresina e Picos – PI.

**Tabela 2:** Entrevistas realizadas, domicílios visitados e taxa de “não-resposta” (TNR) dos domicílios segundo unidade primária de amostragem (UPA) em Teresina e em Picos. Piauí, ISAD-PI, 2019.

	Teresina					Picos					
	
	Entrevistas realizadas	Domicílios sorteados	Domicílios entrevistados	% TNR (Ausência)	% TNR Total	Entrevistas realizadas	Domicílios sorteados	Domicílios entrevistados	% TNR (Recusa)	% TNR (Ausência)	% TNR Total
	48	49	15	11,5	42,3	37	43	18	9,3	48,8	58,1
	42	12	19	9,4	40,6	35	33	17	9,1	39,4	48,5
	9	24	5	25,0	68,8	23	27	13	14,8	37,0	51,9
	17	13	7	5,9	35,3	27	28	14	7,1	42,9	50,0
	33	26	15	40,0	40,0	35	32	18	15,6	28,1	43,8
	39	33	15	26,9	42,3	54	38	22	10,5	31,6	42,1
	78	36	30	12,2	38,8	32	26	14	23,1	23,1	46,2
	33	51	12	0,0	0,0	72	37	33	5,4	5,4	10,8
	25	17	11	16,7	54,2	48	25	20	8,3	8,3	16,7
	30	20	13	0,0	0,0	33	26	17	7,7	26,9	34,6
	47	26	15	7,7	42,3	9	12	6	8,3	41,7	50,0
	40	29	18	15,2	45,5	40	30	17	6,7	36,7	43,3
	75	31	24	11,1	33,3	48	28	19	14,3	17,9	32,1
	60	32	25	15,7	51,0	25	10	10	0,0	0,0	0,0
	49	23	17	0,0	0,0	61	35	26	17,1	8,6	25,7
	17	28	11	35,0	45,0	35	34	16	14,7	38,2	52,9
	50	31	20	11,5	23,1	36	17	17	0,0	0,0	0,0
	38	30	15	37,9	48,3	27	32	11	31,3	34,4	65,6
	62	35	20	22,6	35,5	28	29	11	20,7	41,4	62,1
	61	31	20	22,6	38,7	39	30	18	13,8	24,1	37,9
	25	13	9	21,7	60,9	78	37	29	13,5	8,1	21,6
	46	28	15	17,9	46,4	65	43	24	14,0	30,2	44,2
	54	22	17	22,6	45,2	46	20	20	0,0	0,0	0,0
	29	26	16	33,3	46,7	80	45	31	15,6	15,6	31,1
	53	26,8	27	8,6	22,9	-	-	-	-	-	-
	45	49	17	16,1	45,2	-	-	-	-	-	-
	27	12	13	0,0	0,0	-	-	-	-	-	-
	54	24	19	17,9	32,1	-	-	-	-	-	-
	49	13	17	0,0	22,7	-	-	-	-	-	-
	50	26	20	3,8	23,1	-	-	-	-	-	-

Média ± DP	42,8 ± 16,4	26,8 ± 9,3	19,0 ± 5,5			42,2 ± 18,1	29,9 ± 9	18,4 ± 6,6			

Total	1285	804	497		38,2	1013	717	441			38,3

ISAD-PI: Inquérito de Saúde Domiciliar no Piauí; UPA: unidade primária de amostragem; %TR: % taxa de resposta; %TI: % taxa de impedimento.

Os números de domicílios sorteados em Teresina e em Picos (804 e 715, respectivamente; Tabela 2) foram maiores do que os considerados necessários para a realização das entrevistas, previstos na etapa de amostragem (642 e 688, respectivamente). Apesar disso, o número de domicílios entrevistados foi menor que o planejado, considerando a taxa de “não-resposta” total média de 38,2% em Teresina e de 38,3% em Picos, ambas maiores que a prevista, de 10%.

Do total de domicílios em que houve “não-resposta”, em Teresina, 21,64% ocorreu por motivo de recusa dos moradores em participar da pesquisa e 16,54% ocorreu por exclusão do domicílio devido à ausência de moradores após três tentativas. Por sua vez, em Picos, os impedimentos por recusa e ausência do morador em três tentativas contabilizaram 12,45% e 25,87%, respectivamente.

Foram realizadas 24 estimativas de proporção em relação a idade e sexo (Tabela 3) e 48 estimativas de prevalência de indicadores sociais e de saúde (Tabela 4), totalizando 72 estimativas. À exceção da estimativa de proporção de adolescentes do sexo masculino na Amostra-sangue (*Cv-pˆ* = 27,6), as demais estimativas apresentaram *Cv-pˆ* menor que 20%.

**Tabela 3:** Entrevistas realizadas, estimativas de proporção, intervalos de confiança, coeficientes de variação e efeitos de delineamento segundo sexo de adolescentes, adultos e idosos, em Teresina e Picos. Piauí, ISAD-PI, 2019.

	Masculino			Feminino		
	
	n	pˆ (IC95%)	Cv | deff	n	pˆ (IC95%)	Cv (deff)
**Teresina (Amostra Total)**						

Adolescentes (10–19 anos)	92	21,6(16,0–28,6)	14,3 | 2,4)	135	19,3(16,0–23,0)	8,8 | 1,3
Adultos (20–59 anos)	252	59,3(55,3–63,2)	3,3 | 0,7	413	59,0(56,5–61,4)	2,0 | 0,4
Idosos (60+ anos)	81	19,0(14,8–24,1)	12,0 | 1,4	152	21,7(17,7–26,4)	9,8 | 1,9

**Teresina (Amostra–sangue)**						

Adolescentes (10–19 anos)	32	22,7(12,3–38,0)	27,6 | 3,1	51	18,2(14,2–23,0)	11,7 | 0,8
Adultos (20–59 anos)	75	53,2(45,8–60,4)	6,7 | 0,7	167	59,6(54,6–64,5)	4,0 | 0,7
Idosos (60+ anos)	34	24,1(16,1–34,4)	18.6 | 1,5	62	22,1(17,8–27,2)	10,2 | 0,8

**Teresina (Amostra–R24h)**						

Adolescentes (10–19 anos)	52	24,0(17,5–31,9)	14,7 | 1,5	68	17,0(14,2–20,2)	8,6 | 0,6
Adultos (20–59 anos)	120	55,3(50,5–50,0)	4,2 | 0,5	245	61,2(57,2–65,1)	3,1 | 0,6
Idosos (60+ anos)	45	20,7(14,8–28,3)	15,8 | 1,4	87	21,8(17,4–26,9)	10,6 | 1,2

**Picos**						

Adolescentes (10–19 anos)	75	22,1(18,2–26,5)	9,2 | 0,8	80	14,7(12,0–17,8)	9,5 | 0,8
Adultos (20–59 anos)	215	63,2(58,4–67,8)	3,6 | 0,7	368	67,4(62,5–72,0)	3,4 | 1,3
Idosos (60+ anos)	50	14,7(11,0–19,4)	13,9 | 1,1	98	17,9(13,5–23,5)	13,5 | 2,1

ISAD-PI: Inquérito de Saúde do Piauí; M: masculino; F: feminino; n: entrevistas realizadas; *pˆ*: proporção; IC95%: intervalo de confiança de 95%; *Cv*: coeficientes de variação.

**Tabela 4:** Estimativas de prevalência, intervalos de confiança, coeficientes de variação e efeitos de delineamento segundo sexo de adolescentes, adultos e idosos em Teresina e Picos. Piauí, ISAD-PI, 2019.

Indicadores	Masculino		Feminino	
	
	pˆ (IC95%)	Cv(deff )	pˆ (IC95%)	Cv(deff )
**Teresina**				

Adolescentes (10–19anos)				
Autoavaliação de saúde muito boa ou boa	75,6(65,5–83,4)	5,8 | 0,9	64,0(55,1–71,8)	6,4 | 0,9
Já utilizou o programa de saúde da família	78,9(67,4–87,1)	6,1 | 1,2	84,9(77,5–90,3)	3,6 | 1,9
Aferiu a pressão arterial nos últimos 6 meses	35,6(26,6–46,6)	13,3 | 0,9	38,3(28,7–48,9)	13,0 | 1,4
Autodeclarado preto ou pardo	75,5(64,5–84,0)	6,3 | 1,1	80,5(74,2–85,5)	3,4 | 0,6
Adultos (20–59 anos)				
Auto avaliação de saúde muito boa ou boa	64,5(57,4–71,0)	5,2 | 1,2	55,5(29,9–42,6)	4,0 | 0,8
Já utilizou o programa de saúde da família	64,5(57,3–71,1)	5,2 | 1,2	79,6(73,3–84,7)	3,5 | 1,9
Aferiu a pressão arterial nos últimos 6 meses	62,0(56,0–67,8)	4,7 | 0,9	66,7(61,3–71,6)	3,8 | 1,2
Autodeclarado preto ou pardo	79,2(72,8–84,4)	3,6 | 1,2	77,4(72,8–81,3)	2,7 | 0,9
Idosos (60+ anos)				
Auto avaliação de saúde muito boa ou boa	46,2(35,8–56,8)	11,3 | 0,8	30,4(22,6–39,6)	13,9 | 1,2
Já utilizou o programa de saúde da família	79,5(62,5–90,0)	8,5 | 2,0	81,1(71,8–87,8)	4,8 | 1,5
Aferiu a pressão arterial nos últimos 6 meses	79,5(70,3–86,4)	4,9 | 0,7	90,4(84,8–94,2)	2,5 | 0,9
Autodeclarado preto ou pardo	82,0(69,0–90,0)	6,3 | 1,4	77,0(67,9–84,2)	5,2 | 1,3

**Picos**				

Adolescentes (10–19anos)				
Auto avaliação de saúde muito boa ou boa	82,4(70,7–90)	5,6 | 1,0	80,5(67,2–89,3)	6,6 | 1,4
Já utilizou o programa de saúde da família	76,5(67,9–83,3)	4,8 | 0,5	84,4(75,2–90,6)	4,4 | 0,8
Aferiu a pressão arterial nos últimos 6 meses	33,8(22,6–47,3)	17,9 | 1,1	33,8(22,4–44,6)	14,6 | 0,8
Autodeclarado preto ou pardo	57,4(44,6–69,2)	10,6 | 1,0	67,5(54,1–78,6)	8,9 | 1,3
Adultos (20–59 anos)				
Auto avaliação de saúde muito boa ou boa	67,3(61,7–72,5)	3,9 | 0,6	55,1(49,2–61,0)	5,2 | 1,2
Já utilizou o programa de saúde da família	60,5(53,0–67,5)	5,9 | 1,1	87,2(83,0–90,5)	2,0 | 1,0
Aferiu a pressão arterial nos últimos 6 meses	56,1(50,2–61,8)	5,0 | 0,7	70,7(63,577,1)	4,7 | 1,8
Autodeclarado preto ou pardo	69,3(60,2–77,1)	6,0 | 1,6	71,6(64,0–78,1)	4,8 | 2,0
Idosos (60+ anos)				
Auto avaliação de saúde muito boa ou boa	47,9(33,7–62,5)	14,9 | 1,0	44,8(33,1–57,0)	13,2 | 1,3
Já utilizou o programa de saúde da família	81,3(70,1–90,0)	5,6 | 0,6	89,6(74,1–96,3)	5,5 | 2,5
Aferiu a pressão arterial nos últimos 6 meses	80,9(69,1–88,9)	5,9 | 0,7	81,1(71,2–88,1)	5,0 | 1,0
Autodeclarado preto ou pardo	66,7(50,8–79,5)	10,6 | 1,1	65,6(54,9–75,0)	7,5 | 1,0

ISAD-PI: Inquérito de Saúde Domiciliar no Piauí; M: masculino; F: feminino; *pˆ*: proporção; IC95%: intervalo de confiança de 95%; Cv: coeficiente de variação do erro-padrão; *deff*: efeito de delineamento.

O efeito do delineamento da proporção em relação a sexo e idade (Tabela 3) foi menor que 1,5 para 20 das 24 estimativas. As estimativas de proporção que apresentaram *deff* maior que 1,5 foram proporção de idosos do sexo feminino em Teresina (*deff* = 1,9) e em Picos (*deff =* 2,1), bem como proporção de adolescentes do sexo masculino em Teresina (*deff* = 2,4) e proporção de adolescentes do sexo masculino na Amostra-sangue.

Em relação às estimativas de prevalência dos indicadores sociais e de saúde (Tabela 4), o efeito do delineamento foi menor que 1,5 em 87,5% das estimativas (42 das 48 estimativas) e menor que 2 em 95,8% das estimativas (46 das 48 estimativas).

## DISCUSSÃO

O ISAD-PI destaca-se por ser um estudo populacional domiciliar, que aborda determinantes e condições de saúde em duas cidades do Piauí e abrange vários componentes da saúde. Com isso, espera-se obter dados e indicadores em saúde mais específicos e detalhados, que atendam às necessidades em nível municipal e regional, além de contribuir com informações relevantes para pesquisas nacionais.

O Brasil é um país extenso, com grande diversidade socioeconômica^9^, de forma que é fundamental que inquéritos municipais como o ISAD-PI sejam realizados. O Piauí tem IDH (Índice de Desenvolvimento Humano) de 0,646 e é um dos estados brasileiros com menor índice, superior apenas aos do Maranhão (0,639) e de Alagoas (0,631)^8^. Assim, cidades do Piauí possuem uma realidade diferente de muitas cidades no Brasil. Ademais, a decisão de incluir no estudo a capital, Teresina-PI (IDH: 0,751) e uma cidade do interior do estado, Picos-PI (IDH:0,698), favorece a avaliação de diferentes realidades dentro do estado.

Em relação aos procedimentos de coleta de dados, o ISAD-PI levou 18 meses para ser concluído, o que pode ser considerado uma desvantagem em relação aos custos da pesquisa. A coleta de informações detalhadas sobre saúde e estilo de vida, assim como a diversidade de medidas antropométricas realizadas em todos os moradores do domicílio, foi priorizada em detrimento da redução de tempo e custo, entretanto, isso permitiu um aprofundamento das informações de saúde geradas.

O número de domicílios sorteados foi maior que o concebido na etapa de amostragem, principalmente no município de Teresina, fato relacionado ao crescimento do número de domicílios nos setores censitários de Teresina e Picos desde o censo de 2010. O maior número de domicílios amostrados é resultado da aplicação de frações constantes na etapa de sorteio dos domicílios, assim, manteve-se a equiprobabilidade da amostra, que foi proporcionada pelo sorteio com probabilidade proporcional ao tamanho, entretanto, o controle sobre seu tamanho final foi prejudicado.

A participação dos moradores foi menor que a esperada, apesar da utilização de estratégias como divulgação da pesquisa em meios de comunicação, utilização de panfletos informativos e o retorno da equipe aos domicílios mais de uma vez. Muitas recusas deveram-se ao receio dos moradores em abrirem suas casas ou cederem informações para participarem do estudo. A taxa de “não-resposta” verificada neste estudo pode ser utilizada como parâmetro para futuras pesquisas na etapa de planejamento da amostra.

Destaca-se que, embora o número de domicílios e, portanto, de participantes, tenha sido inferior ao esperado, o número de indivíduos na amostra final foi considerado apropriado para estudos em saúde pública, pois possibilitou precisão adequada, com estimativas, em maioria, com *Cv-pˆ* inferiores a 20%. Quando a estratificação é utilizada adequadamente no plano de amostragem, são obtidos ganhos de precisão nas estimativas médias^17^. A decisão de utilizar a estratificação por sexo e faixa etária na etapa de amostragem deste estudo foi satisfatória.

Desenhos amostrais por conglomerados, como o empregado neste estudo, apresentam um risco de imprecisão dos parâmetros estimados. O *deff* mede a relação entre os estimadores calculados conforme o plano amostral proposto e uma amostra casual simples, sendo, portanto, uma estatística comumente utilizada para avaliar esse efeito^18,19^. Neste estudo, os valores de *deff* inferiores a 2 sugerem que a diferença entre os valores obtidos segundo o desenho amostral empregado e uma amostra casual simples não são expressivamente diferentes, indicando que o delineamento da amostra foi bem-sucedido.

A amostra do ISAD-PI gerou estimativas próximas às previstas, considerando que a maioria das estimativas apresentaram *Cv* abaixo de 20% e que, em mais de 90% delas, o *deff* foi menor que 1,5. Outros estudos epidemiológicos na área da saúde têm proposto esse valor como limite aceitável^3,4,7^. Ademais, a precisão verificada no estudo mostra que foi acertada a escolha de manter a autoponderação da amostra, considerando que o sorteio foi equiprobabilístico.

A precisão e o efeito do delineamento, examinados na avaliação das estimativas deste estudo, indicam que os dados gerados a partir do ISAD-PI poderão contribuir para a avaliação das condições de saúde e morbidade na população, bem como para a avaliação e a implantação de serviços e ações em saúde nos municípios avaliados. Aspectos metodológicos empregados nesta pesquisa poderão servir de base para estudos realizados em outras cidades do Brasil.

Entretanto, é importante estar atento à utilização de resultados relacionados a eventos raros, os quais devem ser interpretados com cautela, em particular, quando feitos com amostras pequenas. As poucas estimativas com *deff* maior que 1,5 e *Cv* maior que 20% ocorreram principalmente entre adolescentes e idosos, cujo tamanho amostral foi menor que o estrato de adultos.

## CONSIDERAÇÕES FINAIS

A realização de inquéritos de saúde representativos da população é de grande importância para a avaliação das condições de saúde nos municípios brasileiros, especialmente em cidades do Nordeste. Essa região do país carece de estudos epidemiológicos com métodos robustos de amostragem que garantam a representatividade dos dados. Além disso, é importante destacar que seus hábitos de vida e alimentação guardam particularidades em relação àqueles praticados pelos habitantes das regiões Sul e Sudeste, onde é realizada a maior parte dos inquéritos de saúde.

Este Inquérito foi desenhado com utilização de técnica de amostragem robusta, garantindo a representatividade dos dados. Como inquéritos domiciliares apresentam inúmeros desafios aos pesquisadores, é necessário e importante, para o desenvolvimento de novas pesquisas, que se disponha de subsídios que levem ao aprimoramento dos delineamentos de amostragem e que se discuta estratégias para aprimorá-los.
